# The Impact of COVID-19 on Rural Food Supply and Demand in Australia: Utilising Group Model Building to Identify Retailer and Customer Perspectives

**DOI:** 10.3390/nu13020417

**Published:** 2021-01-28

**Authors:** Jillian Whelan, Andrew Dwight Brown, Lee Coller, Claudia Strugnell, Steven Allender, Laura Alston, Josh Hayward, Julie Brimblecombe, Colin Bell

**Affiliations:** 1 Global Obesity Centre, Institute for Health Transformation, Deakin University, 1 Gheringhap Street, Geelong, VIC 3220, Australia; andrew.brown@deakin.edu.au (A.D.B.); lee.coller@lhpcp.org.au (L.C.); claudia.strugnell@deakin.edu.au (C.S.); steven.allender@deakin.edu.au (S.A.); laura.alston@deakin.edu.au (L.A.); josh.hayward@deakin.edu.au (J.H.); colin.bell@deakin.edu.au (C.B.); 2 Lower Hume Primary Care Partnership, Seymour, VIC 3661, Australia; 3 Department of Nutrition, Dietetics and Food, School of Clinical Sciences, Monash University, Melbourne, VIC 3168, Australia; julie.brimblecombe@monash.edu

**Keywords:** rural food supply, COVID-19, rural health, food security, food supply chain, community-based system dynamics

## Abstract

Prior to the 2020 outbreak of COVID-19, 70% of Australians’ food purchases were from supermarkets. Rural communities experience challenges accessing healthy food, which drives health inequalities. This study explores the impact of COVID-19 on food supply and purchasing behaviour in a rural supermarket. Group model building workshops explored food supply experiences during COVID-19 in a rural Australian community with one supermarket. We asked three supermarket retailers “What are the current drivers of food supply into this supermarket environment?” and, separately, 33 customers: “What are the current drivers of purchases in this supermarket environment?” Causal loop diagrams were co-created with participants in real time with themes drawn afterwards from coded transcripts. Retailers’ experience of COVID-19 included ‘empty shelves’ attributed to media and government messaging, product unavailability, and community fear. Customers reported fear of contracting COVID-19, unavailability of food, and government restrictions resulting in cooking more meals at home, as influences on purchasing behaviour. Supermarket management and customers demonstrated adaptability and resilience to normalise demand and combat reduced supply.

## 1. Introduction

The Lancet Commission on Obesity highlights malnutrition in all its forms (obesity, undernutrition and other dietary risks) as the leading causes of ill health globally [1]. The ability to make profits from and growing demand for processed and ultra-processed foods and high-status foods such as red meat has seen national and international food systems pivot their food systems towards production of these foods, contributing to malnutrition, environmental degradation and growing inequity. As in many developed countries this food system shift began in Australia during the 1960′s and 1970′s [2] triggering increasing rates of obesity [3] and chronic disease as well as environmental damage [1].

Rural Australian’s bear the brunt of these impacts. Adults living in regional/remote areas of Australia experience higher rates of overweight/obesity than those living in major cities [4]; and obesity is related to chronic diseases such as diabetes, heart disease and specific cancers [5]. A recent study showed that if rural Australians met public health recommendations for diet, physical activity, alcohol and smoking, up to 50% of cardiovascular disease mortality could be reduced [6]. Reducing dietary risk factors seems a clear and important avenue to improve the health of current and future generations in rural areas; and the double-burden of socioeconomic deprivation and health damaging environments (known as deprivation amplification) [7].

In Australia, approximately 70% of food is purchased from supermarkets and grocery stores [8]. Australia has a heavily concentrated supermarket structure with three major chains (Woolworths, Coles and Aldi), together holding 77% market share, with just 7.4% of sales represented by Metcash [9]. Metcash is a wholesale distribution and marketing company that provides food for retail to independent businesses [10]. The market share of supermarkets in food retail gives the retailer significant power. For example, over 2/3 of all supermarket shelf space was devoted to discretionary foods in a study conducted on 100 Victorian supermarkets in Australia [11], suggesting significant power to manipulate customer purchasing patterns and vulnerability for customers if the supply chain is disrupted. Because of the limited number of stores, rural communities are dependent on what retailers’ supply and it is more difficult to access healthy food than in urban areas, with healthier options being both more expensive and less available [12,13]. Rural areas often experience the concept of ‘outshopping’, where local residents travel to neighbouring, usually larger, retail centres to access greater variety and/or cheaper prices across a range of products [14]. Limiting the need to outshop may enhance the local economy. A recent review established that there are few initiatives to improve the healthiness of food retail environments in rural areas [15], and research into dietary intake and food environments in rural areas is limited both at the individual and population level [16]. Limited choice also means rural communities are more vulnerable to environmental and social shocks. Environmental disasters such as bushfires, floods and droughts have tested the resilience of the food supply in rural Australia in the past [17], with researchers conceptualizing food chains in terms of four key elements of resilience: scale, diversity, flexibility and cohesion.

Many people were introduced to the 2020 COVID-19 pandemic with media images of empty supermarket shelves in countries around the world [18]. Australia was no exception with many supermarkets selling out of staple pantry items, fresh foods, and household goods due to a rapid increase in demand for products, logistics restrictions (air, road and sea), factory closures overseas, reduced imports, higher biosecurity, labour shortages (locally and overseas) and reduced spending overseas on our exports [19]. The first case of COVID-19 was reported in Melbourne, Victoria in late January, shortly after Australian borders were closed, social distancing rules were imposed, and services deemed ‘non-essential’ were closed or severely curtailed, increasing rates of unemployment. In the first wave of cases (Wave 1), numbers grew rapidly and, on 30 March, there were 821 active cases in Victoria [20]. In March 2020, early government intervention pushed large sectors of the Victorian workforce into “working from home,” and social isolation policies were enacted, limiting social contact [20]. This was followed by a second wave (Wave 2) of cases in Victoria from June to October 2020. These restrictions on movement, social distancing rules in workplaces, some ‘panic buying’, and rising costs of staple foods all impacted food security in Australia during COVID-19 [21] and internationally [22]. To date, the state of Victoria has experienced Australia’s largest outbreak of COVID-19 and a severe lockdown—at the time, one of the most restrictive globally [23]. The dependence of rural communities on a small number of suppliers and retailers and the impact of COVID-19 on the movement of people and availability of food is likely to have had a major impact on the supply and demand of food in rural Australia. In this paper, we aim to identify and connect factors influencing supermarket food supply and customer demand in a rural community of Australia in response to the impacts of COVID-19.

## 2. Materials and Methods

### 2.1. Setting

This study was set in a rural town in Victoria, approximately 150 km from the nearest capital city and where agriculture, forestry, and tourism are the major industries. The population is approximately 2500, with a median age of 51 years (state median of 37). Approximately 12% of the town’s population had completed a bachelor’s degree or higher (state average 24%) and the median family income was 79% of the state-wide median [24]. The town has one supermarket. The nearest larger grocery retailers are approximately 70 km by road, inaccessible to many residents during ‘lockdown’.

### 2.2. Design

This research comprises a qualitative case study and is reported through the use of the COREQ checklist [25] as recommended for the reporting of qualitative studies. Ethics approval for this study was obtained from Deakin University (HEAG-H 20_2020).

### 2.3. Researcher Team and Reflexivity

The research team are members of the Global Obesity Centre at Deakin University recognized for strong community partnerships and co-designing interventions with community, and/or members of the Centre of Research Excellence in Food Retail Environments for Health. Our contribution to this research entailed facilitation gathering retailer and consumer perspectives on the impact of COVID-19 on food supply and demand, provision of expertise in community-based system dynamics and specialised software to identify and connect influences and interpret the findings in the context of academic literature.

### 2.4. Theoretical Framework

Complexity theory underpins this study. The supply of and demand for healthy food in rural areas, particularly in times of crisis, requires consideration of multiple intersecting components within a system and how they change over time [26].

### 2.5. Participants and Recruitment

The supermarket in this study is owned by a private company that manages and operates 17 supermarkets or food retailers, the majority of these are located in Victoria. Researchers emailed the managing director (address available online), inviting participation and requesting the researcher invitation be extended to other senior managers. All of these 17 retailers have a relationship with Metcash [10] as their major food supplier.

Customer participants were recruited, all from one store in the town setting, via in-store flyers, flyers in community venues serving a wide variety of residents from different socio-economic backgrounds, newspaper advertisements and community Facebook pages. Additional participants were recruited via snowball sampling from existing participants. Customers were eligible to attend the workshop if they were over 18 years of age and were regular customers of the supermarket. A $25 non-supermarket voucher was offered as compensation for time to customer participants.

### 2.6. Data Collection

Small workshops were conducted online, and all sessions were recorded and transcribed. The workshop techniques were informed by community-based system dynamics [27] and guided by scripts. The workshops explored food availability and purchasing behaviour change during the pandemic. This information was then collated using software called (Systems Thinking for Community Knowledge Exchange (STICKE) [28] in order to create a visual representation (causal loop diagram or CLD) of the group’s shared understanding of the problem. [27]. Retailers were asked “What are the current drivers of food supply into this supermarket environment?” and customers were asked “What are the current drivers of purchases in this supermarket environment?” (simplified during all workshops to: ‘Why do/did you buy what you buy/bought?’

### 2.7. Data Analysis

The CLD with retailers was created in real time and the transcript was used to refine the CLD. The CLD and explanatory notes were returned to the retailer stakeholder group for comment. CLDs were created with customers in real time and we used NVIVO version 12 [29] to theme transcripts to the food supply resilience framework by Smith, [17] and emergent themes beyond the framework. Transcripts and themes were used to build a joint CLD that was cross-checked against variables from each customer workshop (J.W., L.C.). Differences were discussed (J.W., A.D.B., L.C.) and the CLD was edited to best capture the overall customer perspective. One customer was unable to attend the workshops and provided a written submission. This submission was considered during theming and was cross checked to ensure inclusion in the customer CLD. We include anonymous quotes from retailers and customers to provide further insight on the themes.

## 3. Results

### 3.1. Participants

Three senior managers—the managing director, the operations manager and the buyer/operations manager—from the company participated in the retailer workshop. Thirty-three customers across six customer workshops participated (Table 1).

### 3.2. Supermarket Causal Lool Diagram

**Figure 1:**
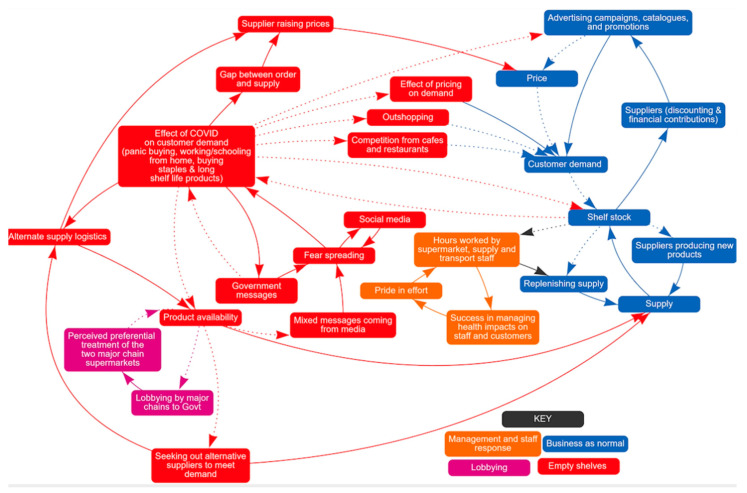
Factors influencing food supply and demand: supermarket retailers’ perspective.

The impact of COVID-19 on food supply and demand from the supermarket retailers’ perspective is shown in Figure 1. Retailers described normal business processes influencing supply and demand and noted the urgency with which they had to respond to COVID-19. Their responses are illustrated as four colour-coded themes: business as normal, empty shelves, lobbying, management and staff response.

#### 3.2.1. Business as Normal (Blue)

In non-pandemic times, the supermarket has reliable and regular access to food to re-stock shelves and meet customer demand. Sometimes suppliers offer incentives to the retailers for certain products that are passed on to customers through promotions or other campaigns influencing price, placement and availability. Suppliers also use promotions to trial new products and capture customer demand. Thus, business as usual involved an equilibrium of customer demand, promotion, purchases, price adjustments and resupply.

#### 3.2.2. Empty Shelves (Red)

With rising numbers of COVID-19 cases and associated restrictions in Victoria, messaging from government, media and social media created and perpetuated fear that food and other items may run out and the town experienced a dramatic and sustained increase in demand for food and other supermarket items that disrupted business as normal.

*‘… so we’ve got our government announcement but you’re very right [manager 2], I think media played a role in that confusion then enhanced that disruption or escalated that disruption.’*

*[manager 1]*

This put pressure on stocks and suppliers were unable to meet demand, so shelves emptied.

*‘Normally when we place an order for say 1000 cases, you’ll get 980 of them … We were down [to] 30% of the number of boxes we were ordering, so if we were ordering 1000 boxes we were getting 300.’*

*[manager 2]*

In response, supermarket managers sought alternate suppliers, and alternate supply logistics (for example, purchased smaller trucks) to boost supply. Managers indicated that there were price increases but, in their experience, this did not dampen demand.

*‘… in the pandemic people were generally less concerned about price, it was just about availability [manager 3]. Specific examples were provided of the extreme price rises to some products: ‘… broccoli went from $35 a box to $100 in one market because there was nothing [else] in there’ and ‘capsicums went from $3 a kilo to about $12, we were selling them at $15′*

*[manager 3]*

Restrictions on numbers of customers in cafes and restaurants caused some of them to close, increasing customer demand for supermarket products. Travel restrictions and fear of being exposed to the virus reduced ‘outshopping’ where community members go to a larger store outside of their local community to ‘stock up’, increasing customer demand on the local supermarket.

*‘… the channels from which you [customers] could get food had diminished, so supermarkets became the main outlets for that and in addition to that and because of the panic buying, people were filling their pantries and buying more product.’*

#### 3.2.3. Lobbying (Purple)

A complicating factor for managers was the buying power and lobbying by major chains to the government to protect their own supplies. They felt that this negatively impacted product availability and their own supply.

*‘There were times when [we] felt like there was definitely preferential treatment to the major chains and whether that was real or imagined.’*

*[manager 1]*

#### 3.2.4. Management and Staff Response (Orange)

Management and key staff worked long hours to source alternate supply, to build a COVID-safe environment both for staff and for customers, leading to a reflection that in an extremely complex and uncertain retail and health environment, this supermarket achieved significant success in maintaining supply and serving customers through the first wave.

*‘we were doing our best and as a small team, we were doing our best’*

*[manager 1]*

### 3.3. Customer Causal Loop Diagram

**Figure 2:**
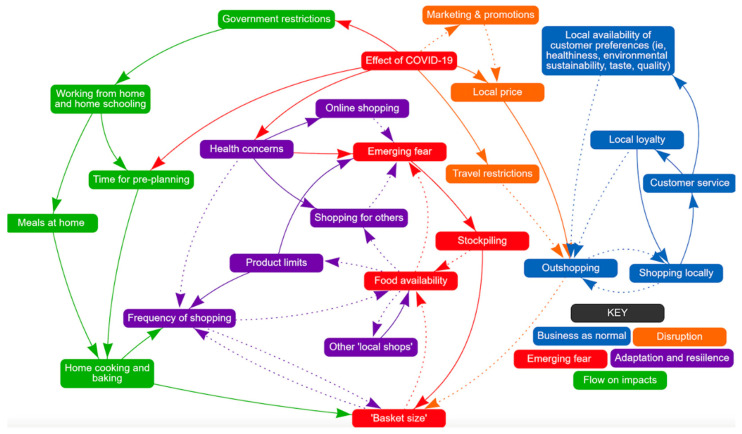
Factors influencing food supply and demand: customers’ perspective.

The impact of COVID-19 on food supply and demand from the customers’ perspective is shown in Figure 2 colour coded into five themes: business as normal, emerging fear, disruption, adaptation and resilience, and flow-on impacts.

#### 3.3.1. Business as Normal (Blue)

Pre-COVID, customers had a range of individual preferences that determined what they purchased, and local availability of those preferences meant that there was limited need to shop outside the town. Customers reported strong local loyalty and good customer service, reinforcing that shopping locally supported the local economy and decreased the need to go elsewhere.

*‘If you don’t use what you’ve got locally, then that will go. You’ve only got one supermarket, so a lot of my decisions about where I shop, I put past that filter’*

*[customer 2]*

Despite this loyalty, there were several customers who reported the need to shop elsewhere, most commonly driven by cheaper prices at larger chains.

#### 3.3.2. Disruption (Orange)

Along with retailers, customers identified price increases as an effect of COVID-19 and it was noticed that marketing and promotions stopped during the initial outbreak. The normal customer response would be to outshop. However, with the travel restrictions and increased fears, consumers continued shopping locally, despite no marketing/promotions and some price increases.

*‘during covid it went straight back up to the $5 price, so it’s not it even that there were no sales, there was like a 30 to 40% jump on the price.’*

*[participant 5]*

#### 3.3.3. Emerging Fear (Red)

Fear of being exposed to COVID-19 or running out of food was at the centre of the joint CLD causing customers to stockpile and increase their ‘basket size’ each time that they went shopping, reducing food availability.

*‘Like I knew we were using the packets of flour we had, so I just bought a spare one just in case. You know it is that stock piling that people tell us not to do, but I only bought one kilo as a back-up stock.’*

*[participant 2]*

#### 3.3.4. Adaptation and Resilience (Purple)

COVID-19 put health concerns at the front of customers minds, particularly those who had underlying conditions, aggravating fear. Product limits (for example, limits on the amount of pasta you could buy on one visit) added to this because it meant customers had to visit the supermarket more often.

*‘it’s covid-19, it’s a choice made about avoiding other people.’*

*[customer 6]*

To adapt, customers moved to online grocery shopping (introduced by this local supermarket during COVID-19) or liaised with friends or family to shop for them, particularly where they had specific dietary requirements and health concerns. Most reported that these strategies reduced their fear and anxiety. Customers also purchased food from other local shops, eg fruit and vegetable store, butchers, bakery but indicated that this strategy on its own did not resolve the overarching issue of insufficient food supply.

#### 3.3.5. Flow on Impacts (Green)

When government restrictions required people to work from home and attend school from home, this increased the number of meals prepared and consumed at home. Some also said that being at home gave them more time for planning and increased their home cooking and baking. This in turn meant a larger basket size and/or increased their frequency of shopping, again reducing food availability.

*‘I think it’s because I had a bit more time and there’s been a lot of talk about food recipes, so it’s probably just motivated me.’*

*[participant 5]*

Finally, some customers reported changing their dietary patterns, as captured in the following quote.

*‘I remember going in when Covid started and there was absolutely none [chocolate] of them were on offer and I suppose that’s good because … you probably shouldn’t eat it, but you know, you’ve got [to] treat yourself sometimes and I noticed that none of them were on offer and I didn’t actually buy any.’*

*[participant 10]*

## 4. Discussion

The COVID-19 pandemic profoundly changed food supply and demand in this rural Australian town. Fear of the disease and of not being able to access food drove this change and adaptability and resilience were displayed by both supermarket management and customers to normalise demand and combat reduced supply. Our findings challenge the rhetoric of ‘panic buying’ as insufficiently nuanced to explain the complexity of the response by customers to the pandemic. In reality people were protecting themselves and/or others from potential illness by having food reserves at home.

Others have reported COVID-19-related stressors impacting rural food supply. National and international responses to the pandemic included restrictions on travel, trade and lockdown of cities similar to those in Victoria that constrained the ability of the food supply to meet customer demand [21,30,31] and impacted the ability of rural and remote communities to access sufficient, affordable and nutritious food [21]. Alongside these constraints on food supply, and consistent with our findings that mixed messages exacerbated food supply problems, Trnka also observed that mixed and mass media messaging intensified psychological stress [32]. COVID-19-related health fears have been acknowledged across multiple human experiences including mental health [33], dentistry [34], and oncology [35], suggesting that this fear and psychological stress could be considered a rational response to an extraordinary occurrence such as a pandemic. Specifically, psychiatrists have explained that what was perceived as ‘panic buying’ or ‘hoarding’ during the pandemic were expected behaviours by people preparing for a known shortage [36].

Our findings support the premise that ‘just-in-time’ food supply chains that minimise on-site stock reserves, although preferred by retailers as a profitable business model in usual circumstances, lack the resilience required when unexpected supply disruptions occur [31]. Retailers in our study report their significant efforts to source alternate suppliers and logistics outside their usual supplier because the continuous product flows on which the just-in-time model is based and as reported in Hobbs (2020) [31] did not withstand the pandemic demand and supply issues.

Further, our findings point to the need for strengthening food supply resilience in rural Australia to survive future extreme events such as natural disasters (e.g., floods) [37] or infectious disease outbreaks such as COVID-19 [31], and present additional unique challenges for vulnerable regional populations and communities. Shorter supply chains may be one way of strengthening food supply resilience. After extreme flooding in Rockhampton, Queensland, in 2011, long supply chains (those typically serving supermarkets in urban and regional areas which rely on interstate transport and rapid restocking) experienced more major disruptions than the shorter supply chains (e.g., local farmers) that were largely able to supply food to the regional community [17]. A recent study of the impacts of COVID-19 on food systems similarly called for small-scale local farmers and shorter food supply chains to sharpen food resilience [18]. Given the complexity we observed in our study of a small rural community, this is likely to be only part of the solution.

The perception of retailers in this study that larger chains received preferential treatment is supported by the literature and may point to another part of the solution—more access to decision-making processes for small grocery stores. Larger retailers, those most likely engaged in these long supply chains, are also those more likely to be engaged with disaster governance and hold stronger links to government, as was the case in this pandemic [17,38]. For example, the Coles Group Ltd., the second largest grocery chain store in Australia [39], led a successful submission to the Australian Competition and Consumer Commission in order to enable large retailers, who were invited to join this action, to by-pass usual competitive processes [40] to obtain food supply. The power to move swiftly to take such action is likely beyond individual retailers in short supply chains.

The residents of the rural community we examined in this study showed resilience in adapting local resources to meet their requirements. In particular, residents adopted multiple strategies to minimise impacts on their existing health problems. This was prudent given the emerging body of evidence that those at higher risk of infection and complications from COVID-19, include people living with hypertension, obesity [41], diabetes [42], co-morbidities and the elderly [43], and these people should be supported to source food safely. Our study identified multiple adaptive strategies that consumers adopted to protect themselves. Customers reported that the introduction of online shopping, people shopping for others and changing the frequency of shopping trips reduced the risk of infection and anxiety. One impact of COVID-19 on the food system, identified in this study, was that more people were working and schooling from home, which led to increased purchases of staple foods and more cooking at home. Emerging evidence suggests that additional time spent at home may improve or detriment people’s diets. A study by Eftimov (2020) [44] compared recipes sourced from the internet pre and during ‘lockdown’. If sourcing recipes can be considered a proxy for diet, consumption of salt, fat, sweets and juice decreased during the quarantine period as home cooking generally contains less of these ingredients than café or restaurant foods. This contrasts to an online cross-sectional survey of 2447 adults in Lithuania by Kriaucionere (2020) [45], which found that almost half of the residents ate more than usual, with 45% snacking more frequently. The 62% of people who reported cooking at home more often acknowledged that they consumed more homemade pastries and fried foods. The same study reported that 61% of these adults reduced their physical activity and 32% indicated that they had gained weight. A study of 7514 Spanish participants who completed on online survey showed a statistically significant improvement in diet score. [46]. Further research is needed to draw more robust conclusions on the short- and long-term impact of COVID-19 on diet.

A strength of this study was that we gathered data from retailers and customers during the COVID-19 pandemic in Australia and we present both perspectives on the impact of the pandemic on food demand and supply. Further, the methodology captures how the impact of COVID-19 changed food supply over time and how the causes and consequences of the pandemic intersect. There are also a number of limitations. We report on a single rural Australian community and the experiences of other communities may have been different. Further, sales data would have added valuable quantitative information to our findings, but it was inappropriate to request such data from our participants during such a busy and stressful time. Socio-demographic information about our customers may have offered further insight into which customers were most affected. However, we elected not to gather this information in order to encourage participation. The views may not reflect those of the complete adult customer population, but they do provide initial insights that warrant further investigation. We acknowledge that the supply chain was put under great stress, and the resilience of Australia’s food supply chain warrants investigation. Finally, we did not assess the longer-term impact of COVID-19 on food demand and supply. For example, a concern expressed by customers was that fruit and vegetable pickers were in short supply. This could lead to future consequences if fruit on trees that were not being picked, leading to less availability of fresh fruit and vegetables and reduced cash flow in the community.

## 5. Conclusions

Fear of exposure to the SARS-COV-2 coronavirus that causes COVID-19 and fear of not being able to access food and household items, along with a supply chain that struggled to re-orient in a tight time frame, disrupted the food system in a rural Australian town. Pre-COVID-19 this community was centred on customer loyalty and the supermarket previously experienced equilibrium between supply and demand. Adaptability and resilience were displayed by both supermarket management and customers in the strategies adopted to normalise demand and combat reduced supply during COVID-19.

Access and availability of food tend to be taken for granted in Australia. The COVID-19 pandemic challenged this view and exposed weaknesses in the food supply chain in Victoria for a rural community that was already vulnerable to food supply and related health issues. Altieri et al. (2020) have argued that transition to more socially just, ecologically resilient, localized food systems is urgently needed [30] and it is our hope that the crisis of COVID-19 brings urgency to re-imagining food systems so that they are more local, more seasonal and more resilient. The methods used in this study could help retailers and communities work together to design a more resilient food system. Future studies to track the return to a ‘COVID-normal’ food supply, impacts of future ‘waves’ of the virus (if they occur), and detailed studies of the similarities of pandemic shocks with other shocks are required to understand and strengthen long-term resilience in rural food supply.

## Figures and Tables

**Table 1:** Dates of workshops and participant information.

Workshop Date	Management (M) or Customer (C)	Participants (*n*)	Male/Female (*n*/*n*)
1 June 2020	M	3	(3/0)
22 June 2020	C	2	(0/2)
26 June 2020	C	1	(0/1)
1 July 2020	C	5	(1/4)
13 July 2020	C	2	(0/2)
16 July 2020	C	14	(9/5)
30 July 2020	C	8	(3/5)
Written submission	C	1	(0/1)
Total		36	(16/20)

## Data Availability

Data available on reasonable request from the corresponding author. The data are not publicly available due to the small community and number of participants.
